# miR-29b regulates expression of collagens I and III in chondrogenically differentiating BMSC in an osteoarthritic environment

**DOI:** 10.1038/s41598-017-13567-x

**Published:** 2017-10-16

**Authors:** Ute Mayer, Achim Benditz, Susanne Grässel

**Affiliations:** 1 Department Orthopaedic Surgery, Exp. Orthopaedics, ZMB/Biopark 1, University of Regensburg, Regensburg, Germany; 2 Department Orthopaedic Surgery, Asklepiosklinikum, Bad Abbach, Germany

## Abstract

Osteoarthritis (OA) is characterized by a slowly progressing, irreversible loss of articular cartilage. Tissue engineering approaches for cartilage regeneration include stem cell-based strategies but not much is known about their repair capacity in an OA microenvironment. The aim of the present study was to identify factors regulating collagen expression during chondrogenic differentiation of bone marrow-derived mesenchymal stem cells (BMSC) in an OA microenvironment. Coculture with OA cartilage induced miR-29b expression in BMSC which inhibited collagen I and III expression. Elevated miR-29b expression resulted in higher caspase 3/7 activity and promoted apoptosis of BMSC in part by directly inhibiting the anti-apoptotic proteins Bcl-2 and Mcl-1. Stimulation with IFN-γ induced miR-29b expression in BMSC. Our results suggest that miR-29b affects BMSC-based OA cartilage regeneration because expression of collagen III, mainly produced by undifferentiated BMSC, and collagen I, a marker for dedifferentiated chondrocytes, are inhibited by miR-29b thus influencing composition of the newly formed ECM. This might be critical to avoid formation of inferior fibrocartilage instead of hyaline cartilage. Furthermore, higher miR-29b expression promotes apoptosis either preventing excessive cell growth or reducing the number of BMSC undergoing chondrogenesis. Thus, miR-29b has both supportive but possibly also unfavourable effects on BMSC-based OA cartilage regeneration.

## Introduction

Osteoarthritis (OA) is a common, multi-factorial, slowly progressing and irreversible degenerative disorder affecting entire synovial joints mostly of hands, knees and hips. The risk to develop OA increases with age and genetic predisposition, obesity, mechanical influences and traumatic joint injury are other risk factors. OA is characterized by loss of articular cartilage, changes in subchondral bone and variable degrees of synovial inflammation which leads to pain, stiffness, limitation in motility and eventually loss of joint function^1–3^.

Cartilage possesses only very limited capacity for self-repair because it is a non-vascularised, aneural tissue with a low density of cells which are trapped within a tight extracellular matrix^4,5^. Hence, regeneration of degraded cartilage is challenging^6^. Tissue engineering approaches for cartilage regeneration include cell-based strategies using chondrocytes or multipotent stem cells, alone or in combination with scaffolds^7^. Cultured human bone marrow-derived mesenchymal stem cells (BMSC) possess chondrogenic differentiation potential^8^ but not much is known about their capacity to form cartilage under the influence of the OA microenvironment if they are isolated from OA patients, expanded *in vitro* and then reimplanted into a diseased joint. Some studies reported that mesenchymal stem cells (MSC) from patients with OA showed reduced chondrogenic activity^9^ whereas others observed similar chondrogenic potential of MSC from OA patients compared to that of healthy donors^10^ and that the chondrogenic potential of adult MSC is independent of age or OA aetiology^11^. Even if autologous MSC from OA patients may be a promising cell source with chondrogenic differentiation potential, not much is known about the capacity of MSC for chondral or osteochondral regeneration in an OA environment.

The goal of cell-based therapies in OA-pathology is to implant cells into the diseased cartilage area or lesion where they are supposed to form a long-lasting and functional repair tissue. However, the cellular microenvironment in a joint and their paracrine signals can profoundly influence chondrogenic differentiation of MSC^12^. There are both positive and negative effects of OA-related factors on MSC differentiation reported. On the one hand, soluble morphogens secreted by primary OA chondrocytes induced chondrogenic and downregulated hypertrophic differentiation of MSC^13^. On the other hand, catabolic factors present in OA joints inhibited chondrogenesis^14^. In previous studies we observed that articular cartilage and subchondral bone explants from OA patients affected the extracellular matrix (ECM) production and composition of cocultured BMSC^15,16^. In general, coculture with OA cartilage explants resulted in reduced collagen gene and protein expression in chondrogenically differentiating BMSC compared to monocultured cells. Collagen II accounts for up to 95% of the articular cartilage collagens^17^, whereas induction of collagen I synthesis is associated with dedifferentiating chondrocytes losing their chondrogenic phenotype^18^. Collagen I is detected in late-stage OA cartilage^19^ and collagen III is not only detected in OA cartilage^20^ but also in normal cartilage^21^. Undifferentiated BMSC secrete more collagen III than collagen I^22^. If BMSC undergo chondrogenic differentiation in coculture with OA cartilage explants, both collagen I and III expression become reduced compared to BMSC kept in monocultures^15^.

One possible mechanism for the downregulation of collagen I and III expression found in BMSC cocultured with OA cartilage might be via microRNAs (miR), short non-coding RNAs which regulate gene expression post-transcriptionally^23^. MiRs play a role in normal cartilage matrix development and homeostasis participating in the regulation of ECM production and turnover, growth factor regulation, differentiation, proliferation and apoptosis of chondrocytes and cartilage degeneration during OA^24–26^. MiRs are implicated in chondrogenesis by modulating expression of growth- and transcription factors and they are involved in the development of OA by regulating proteolytic enzymes^27,28^. During chondrogenic differentiation, MSC alter their miR expression profile: some miRs are upregulated while others are downregulated and many putative targets of these miRs are genes well known to be involved in chondrogenic differentiation^29–32^.

It is reported that SOX9, the master transcription factor of chondrogenesis, represses expression of miR-29a-3p and miR-29b-3p via the 29a/b1 promoter whereas IL-1β increases the expression of the miR-29 family in primary human chondrocytes^33^. Among the multiple putative targets of the miR-29 family are several collagens as predicted by different algorithms using miRWalk2.0^34^. Direct binding of miR-29b to the mRNA within the 3′-UTR of COL1A1 and COL3A1 leading to mRNA degradation or preventing its translation is confirmed by luciferase reporter gene assays e.g. by Steele, *et al*.^35^.

In a previous study we observed that both collagen I and III expression was downregulated in BMSC undergoing chondrogenic differentiation in coculture with OA cartilage explants compared to BMSC kept in monocultures^15^. The aim of the present study was to identify factors of the OA microenvironment which are responsible for the regulation of collagen I and III expression during chondrogenic differentiation of BMSC in coculture with OA cartilage. We hypothesized a miR-mediated regulation of collagen I and III expression. Hence, we investigated the influence of OA cartilage on the expression of a specific miR, miR-29b-3p, in cocultured chondrogenically differentiating BMSC. In addition, we analysed the effects of miR-29b-3p overexpression and inhibition on the expression of its targets, COL1A1 and COL3A1 and its influence on cell survival. Furthermore, BMSC were stimulated with soluble factors known to be released from OA cartilage to identify a candidate molecule responsible for miR-29b-3p regulation.

## Results

Two different 3D culture systems to induce chondrogenic differentiation of BMSC were used and some experiments were performed in 2D monolayer culture as stated in the figure headings. For direct coculture with OA cartilage/bone, BMSC were embedded in fibrin gels and indirect coculture with medium conditioned with OA cartilage was performed with micromass pellets.

### Soluble OA cartilage derived factors promote miR-29b expression in cocultured BMSC and repress *COL1A1* and *COL3A1* gene expression and protein secretion

**Figure 1:**
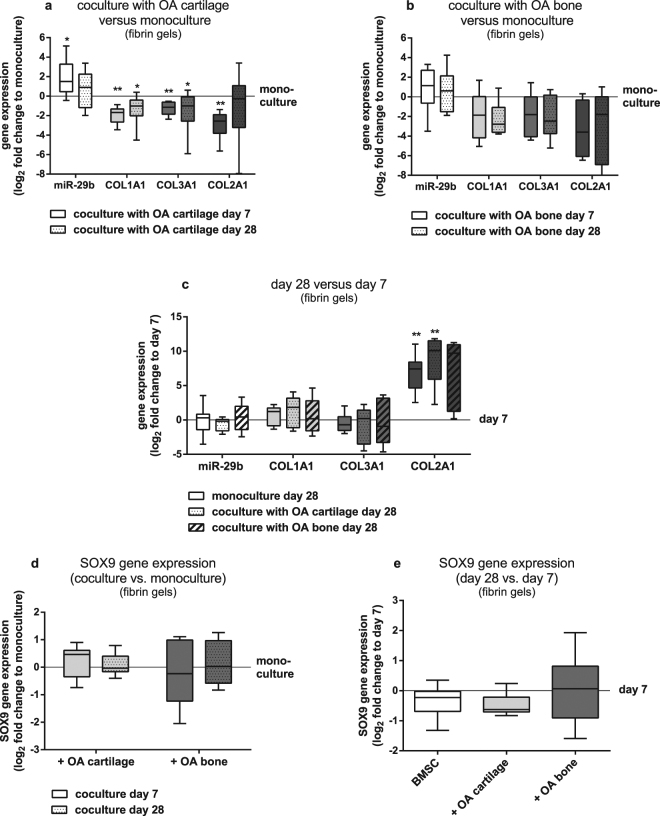
MiR-29b, *COL1A1*, *COL2A1*, *COL3A1* and *SOX9* gene expression of BMSC cocultured with OA cartilage and OA bone explants. (**a**) miR-29b (white bars), *COL1A1* (light grey bars), *COL3A1* (dark grey bars) and *COL2A1* (black bars) gene expression of BMSC embedded in fibrin gels and cocultured on OA cartilage explants for 7 days (bars without pattern) and 28 days (bars with pattern) compared to corresponding monocultures (zero line, n = 8). (**b**) miR-29b (white bars), *COL1A1* (light grey bars), *COL3A1* (dark grey bars) and *COL2A1* (black bars) gene expression of BMSC embedded in fibrin gels and cocultured on OA bone explants for 7 days (bars without pattern) and 28 days (bars with pattern) compared to corresponding monocultures (zero line, n = 6). (**c**) miR-29b (white bars), *COL1A1* (light grey bars), *COL3A1* (dark grey bars) and *COL2A1* (black bars) gene expression of BMSC embedded in fibrin gels in monoculture (bars without pattern), in coculture with OA cartilage explants (dotted bars) and in coculture with OA bone explants (hatched bars) at day 28 compared to day 7 (zero line, n = 6–8). (**d**) *SOX9* gene expression of BMSC embedded in fibrin gels and cocultured on OA cartilage (light grey bars) and OA bone explants (dark grey bars) for 7 days (bars without pattern) and 28 days (bars with pattern) compared to corresponding monocultures (zero line; n = 6). (**e**) *SOX9* gene expression of BMSC embedded in fibrin gels in monoculture (white bar), in coculture with OA cartilage explants (light grey bar) and in coculture with OA bone explants (dark grey bar) at day 28 compared to day 7 (zero line, n = 6). Results are expressed as box plots with median, the 25^th^ and 75^th^ percentiles and whiskers showing the largest and smallest values. * = p < 0.05; ** = p < 0.01; non-parametric Wilcoxon signed rank test for paired analysis.

BMSC embedded in fibrin gels subjected to chondrogenic differentiation on top of OA cartilage explants for 7 and 28 days, exhibited lower *COL1A1* and *COL3A1* and higher gene expression levels of mature miR-29b-3p compared to BMSC kept in monoculture without cartilage explants (Fig. 1a). *COL2A1* gene expression was significantly lower in cocultured BMSC compared to monocultures at day 7. OA subchondral bone explants had no significant effect on miR-29b, *COL1A1*, *COL3A1* and *COL2A1* gene expression of cocultured BMSC subjected to chondrogenic differentiation after 7 and 28 days compared to monocultured cells (Fig. 1b). In both, BMSC kept in monoculture and coculture with OA cartilage *COL2A1* gene expression increased from day 7 to day 28 indicating chondrogenic differentiation whereas no significant change in *COL2A1* expression of BMSC in coculture with OA subchondral bone occurred at day 28 compared to day 7. Notably, miR-29b, *COL1A1* and *COL3A1* gene expression remained unaltered in all culture conditions until 28 days compared to day 7 (Fig. 1c).

As Le *et al*. observed an inverse correlation of miR-29 and *SOX9* gene expression during chondrogenesis of MSC and showed that Sox9 overexpression repressed expression of miR-29 family members, whilst knockdown of Sox9 increased their expression^33^, we analysed *SOX9* gene expression level of BMSC cocultured with OA cartilage and bone. After 7 and 28 days of chondrogenic differentiation of BMSC embedded in fibrin gels and cultured on top of OA cartilage- and bone explants we did not detect changes in *SOX9* expression (Fig. 1d). Compared to day 7, *SOX9* gene expression remained unaltered in BMSC in monoculture and in coculture with OA cartilage and bone explants until the end of culture at 28 days (Fig. 1e).

**Figure 2:**
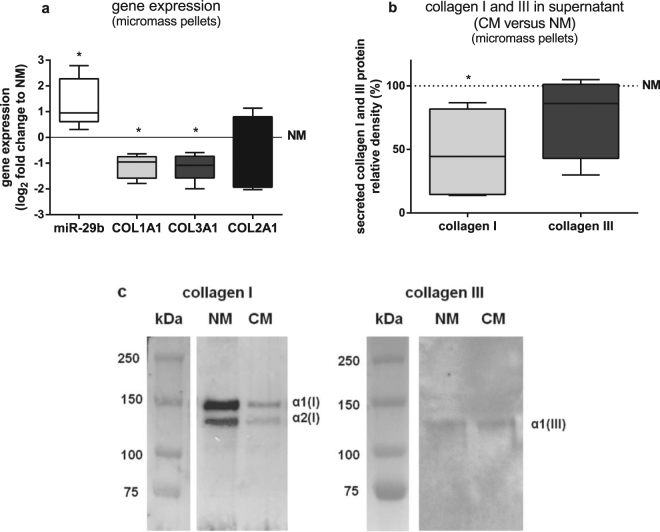
Gene expression and protein secretion of BMSC cultured in medium conditioned with OA cartilage. (**a**) miR-29b (white bar), *COL1A1* (light grey bar), *COL3A1* (dark grey bar) and *COL2A1* (black bar) gene expression of BMSC kept in micromass pellets and cultured for 7 days in chondrogenic medium conditioned with OA cartilage (CM) compared to non-conditioned chondrogenic medium (NM, zero line, n = 7). (**b**) Densitometric quantification of alpha1 and alpha2 chains of collagen I (light grey bar) and III (dark grey bar) western blot images of collagen extracts from cell culture supernatants of BMSC kept in micromass pellets for 7 days in CM relative to NM (line at 100%, n = 6). (**c**) Representative collagen I and III western blot images from collagen extracts of cell culture supernatants of BMSC kept in micromass pellets for 7 days in NM and CM (n = 6). Full-length blots are presented in Supplementary Figure S1. Results are expressed as box plots with median, the 25^th^ and 75^th^ percentiles and whiskers showing the largest and smallest values. * = p < 0.05; non-parametric Wilcoxon signed rank test for paired analysis. CM: with OA cartilage conditioned medium; NM: non-conditioned medium.

To determine if the upregulation of miR-29b gene expression and concomitant downregulation of *COL1A1* and *COL3A1* gene expression in BMSC cocultured with OA cartilage is mediated through soluble factors derived from OA cartilage or if a direct cellular contact to OA cartilage is required, we used conditioned culture supernatant from cell-free OA cartilage explants. For that, expression levels of *COL1A1*, *COL3A1*, *COL2A1* and miR-29b of BMSC kept in micromass pellets cultured for 7 days in chondrogenic medium conditioned with OA cartilage (conditioned medium = CM) were compared to BMSC micromass pellets cultured in non-conditioned chondrogenic medium (non-conditioned medium = NM). BMSC cultured in CM expressed significantly more miR-29b and less *COL1A1* and *COL3A1* mRNA than BMSC cultured in NM while *COL2A1* expression was not significantly changed by CM (Fig. 2a). BMSC cultured in CM secreted approximately 50% less collagen I into culture supernatants than BMSC cultured in NM while secretion of collagen III -which could hardly be detected- was not influenced by CM after 7 days (Fig. 2b,c). Collagen II was not detected in supernatants of BMSC micromass pellets after 7 days of chondrogenic induction (data not shown).

### miR-29b regulates *COL1A1* and *COL3A1* mRNA and corresponding protein synthesis

**Figure 3:**
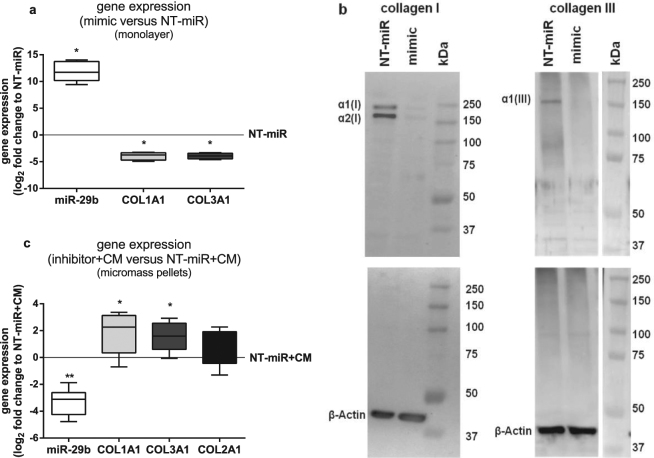
Gene and protein expression of collagens I and III in BMSC transfected with miR-29b mimic and inhibitor. (**a**) miR-29b (white bar), *COL1A1* (light grey bar) and *COL3A1* (dark grey bar) gene expression of BMSC in monolayer transfected with miR-29b mimic compared to BMSC transfected with a non-targeting control miR (NT-miR, zero line, n = 6). (**b**) Representative collagen I (n = 6) and III (n = 3) western blot images of total protein lysates of BMSC in monolayer transfected with mimic and NT-miR and corresponding β-Actin blots as loading controls. (**c**) miR-29b (white bar), *COL1A1* (light grey bar), *COL3A1* (dark grey bar) and *COL2A1* (black bar) gene expression of BMSC transfected in monolayer with miR-29b inhibitor and subsequently cultured in micromass pellets for 7 days in chondrogenic medium conditioned with OA cartilage (CM) compared to BMSC transfected with NT-miR and cultured in CM (NT-miR + CM, zero line, n = 8). Results are expressed as box plots with median, the 25^th^ and 75^th^ percentiles and whiskers showing the largest and smallest values. * = p < 0.05; ** = p < 0.01; non-parametric Wilcoxon signed rank test for paired analysis. mimic: miR-29b mimic; NT-miR: non-targeting control miR; inhibitor + CM: with miR-29b inhibitor transfected BMSC cultured in medium conditioned with OA cartilage; NT-miR + CM: with non-targeting control miR transfected BMSC cultured in medium conditioned with OA cartilage.

To verify if the elevated miR-29b gene expression levels detected in BMSC cocultured with OA cartilage explants or cultured in OA cartilage CM are responsible for the reduced *COL1A1* and *COL3A1* gene expression, miR-29b was overexpressed in BMSC by transfection with a miR-29b mimic, a small, chemically modified double-stranded RNA that mimics the endogenous miR. After transfection, elevated miR-29b levels and accordingly reduced *COL1A1* and *COL3A1* gene expression (Fig. 3a) and corresponding protein synthesis (Fig. 3b) was detected in comparison to that of BMSC transfected with a non-targeting control miR (NT-miR). *COL2A1* gene and protein expression was not detectable in these miR-29b mimic transfected BMSC kept in chondrogenic medium for 2 days. To further confirm that higher miR-29b levels contribute to the reduction of *COL1A1* and *COL3A1* gene expression in BMSC cocultured with OA cartilage or cultured in OA cartilage CM, BMSC were transfected with NT-miR or miR-29b inhibitor. Latter is a small, chemically modified single-stranded RNA molecule designed to specifically bind to and inhibit endogenous miR-29b molecules leading to downregulation of miR activity by preventing the binding of the miR to its target mRNA. The miR-29b inhibitor had no significant influence on collagen expression of BMSC in monolayer culture 72 h after transfection (data not shown) presumably because the impact of inhibiting basal miR-29b levels was too weak to show a detectable effect on collagen protein levels. Therefore, we switched from monolayer to micromass pellet culture kept in CM because that culture regimen resulted in upregulation of miR-29b whose activity could be subsequently blocked by the miR-29b inhibitor. After transfection, BMSC were cultivated as micromass pellets for 7 days in chondrogenic CM. MiR-29b inhibitor transfected BMSC exhibited significantly lower miR-29b and higher *COL1A1* and *COL3A1* gene expression levels compared to BMSC transfected with NT-miR while *COL2A1* gene expression remained unaltered. In NT-miR transfected BMSC the increased miR-29b expression levels, induced by culture in CM, are not blocked (Fig. 3c).

### miR-29b promotes apoptosis of BMSC

**Figure 4:**
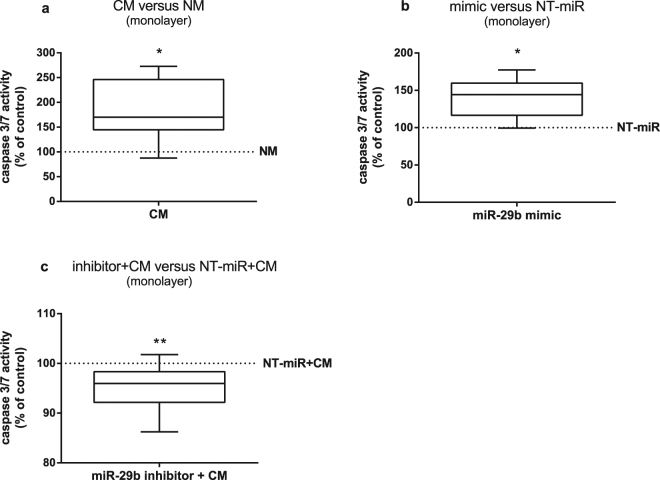
Quantification of caspase 3/7 activity in BMSC. (**a**) Caspase 3/7 activity of BMSC in monolayer cultured in chondrogenic medium conditioned with OA cartilage (CM) relative to non-conditioned medium (NM, line at 100%, n = 8). (**b**) Caspase 3/7 activity of BMSC in monolayer transfected with miR-29b mimic relative to BMSC transfected with a non-targeting control miR (NT-miR, line at 100%, n = 8). (**c**) Caspase 3/7 activity of BMSC in monolayer transfected with miR-29b inhibitor and subsequently cultured in CM relative to BMSC transfected with NT-miR and cultured in CM (NT-miR + CM, line at 100%, n = 10). Results are expressed as box plots with median, the 25^th^ and 75^th^ percentiles and whiskers showing the largest and smallest values. * = p < 0.05; ** = p < 0.01; non-parametric Wilcoxon signed rank test for paired analysis. CM: with OA cartilage conditioned medium; NM: non-conditioned medium; mimic: miR-29b mimic; NT-miR: non-targeting control miR; inhibitor + CM: with miR-29b inhibitor transfected BMSC cultured in medium conditioned with OA cartilage; NT-miR + CM: with non-targeting control miR transfected BMSC cultured in medium conditioned with OA cartilage.

Additionally to its inhibitory effect on collagen expression, miR-29b modulated cell survival. BMSC cultured in CM showed higher caspase 3/7 activity which correlates with increased apoptotic activity compared to that of BMSC in NM (Fig. 4a). Likewise, miR-29b overexpression by transfection of BMSC with miR-29b mimic resulted in higher caspase 3/7 activity compared to BMSC transfected with NT-miR (Fig. 4b). Verifying the contribution of elevated miR-29b expression induced by CM, caspase 3/7 activity in BMSC transfected either with a miR-29b inhibitor or NT-miR and subsequently cultured in CM, was measured. Blocking the activity of miR-29b with the inhibitor significantly reduced caspase 3/7 activity compared to BMSC transfected with a NT-miR (Fig. 4c).

**Figure 5:**
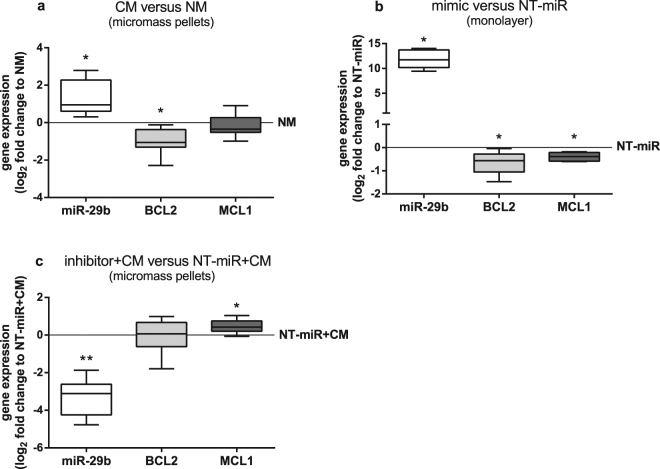
Expression of genes related to apoptosis in BMSC. (**a**) miR-29b (white bar), *BCL2* (light grey bar) and *MCL1* (dark grey bar) gene expression of BMSC kept in micromass pellets and cultured for 7 days in chondrogenic medium conditioned with OA cartilage (CM) compared to non-conditioned chondrogenic medium (NM, zero line, n = 7). (**b**) miR-29b (white bar), *BCL2* (light grey bar) and *MCL1* (dark grey bar) gene expression of BMSC in monolayer transfected with miR-29b mimic compared to BMSC transfected with a non-targeting control miR (NT-miR, zero line, n = 6). (**c**) miR-29b (white bar), *BCL2* (light grey bar) and *MCL1* (dark grey bar) gene expression of BMSC transfected in monolayer with miR-29b inhibitor and subsequently kept in micromass pellets and cultured for 7 days in CM compared to BMSC transfected with NT-miR and cultured in CM (NT-miR + CM, zero line, n = 8). Results are expressed as box plots with median, the 25^th^ and 75^th^ percentiles and whiskers showing the largest and smallest values. * = p < 0.05; ** = p < 0.01; non-parametric Wilcoxon signed rank test for paired analysis. CM: with OA cartilage conditioned medium; NM: non-conditioned medium; mimic: miR-29b mimic; NT-miR: non-targeting control miR; inhibitor + CM: with miR-29b inhibitor transfected BMSC cultured in medium conditioned with OA cartilage; NT-miR + CM: with non-targeting control miR transfected BMSC cultured in medium conditioned with OA cartilage.

A possible mechanism how miR-29b influences caspase 3/7 activity is by modulating expression of regulators of apoptosis like the anti-apoptotic proteins Bcl-2 and Mcl-1, both known targets of the miR-29 family. Culture in CM leads to significantly lower *BCL2* expression in comparison to that of BMSC cultured in NM while *MCL1* expression is unaltered (Fig. 5a). Overexpression of miR-29b by transfection with miR-29b mimic resulted in higher miR-29b level and both *BCL2* and *MCL1* gene expression reduction (Fig. 5b). Transfection with a miR-29b inhibitor decreased miR-29b level and increased *MCL1* gene expression significantly while *BCL2* gene expression remains unaltered compared to the level of expression in BMSC transfected with NT-miR and cultured in CM (Fig. 5c).

### miR-29b does not modulate proliferation of BMSC

BMSC cultured in CM have significantly more BrdU incorporated during DNA synthesis indicating higher proliferation rates compared to BMSC in NM (Supplementary Figure S2a). However, miR-29b overexpression by transfection of BMSC with miR-29b mimic resulted in unchanged BrdU incorporation levels compared to BMSC transfected with a NT-miR (Supplementary Figure S2b).

### Stimulation with IFN-γ induces miR-29b expression

**Figure 6:**
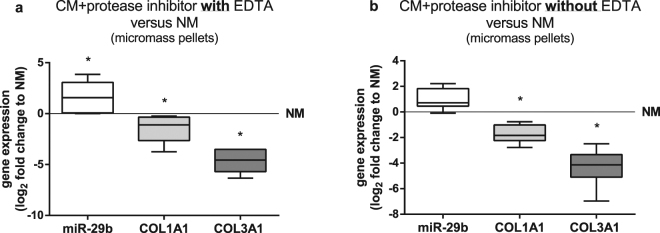
Gene expression of BMSC cultured in conditioned medium supplemented with protease inhibitors. miR-29b (white bars), *COL1A1* (light grey bars) and *COL3A1* (dark grey bars) gene expression of BMSC kept in micromass pellets and cultured for 7 days in chondrogenic medium conditioned with OA cartilage (CM) and supplemented with protease inhibitor cocktail including EDTA which additionally inhibits matrix metalloproteases (**a**) or without EDTA (**b**) compared to non-conditioned chondrogenic medium (NM, zero line, n = 7). Results are expressed as box plots with median, the 25^th^ and 75^th^ percentiles and whiskers showing the largest and smallest values. * = p < 0.05; non-parametric Wilcoxon signed rank test for paired analysis. CM: with OA cartilage conditioned medium; NM: non-conditioned medium.

As soluble factors derived from OA cartilage are responsible for the upregulation of miR-29b expression we tested if the upregulation of miR-29b could be prevented by blocking the activity of proteases in CM using protease inhibitors. Additionally to serine and cysteine proteases, the applied inhibitor cocktail containing EDTA inhibits also matrix metalloproteases (MMPs). Both protease inhibitor cocktails (+EDTA; Fig. 6a and - EDTA; Fig. 6b) did not prevent the upregulation of miR-29b and downregulation of *COL1A1* and *COL3A1* gene expression in BMSC cultured in CM compared to BMSC kept in NM.

**Figure 7:**
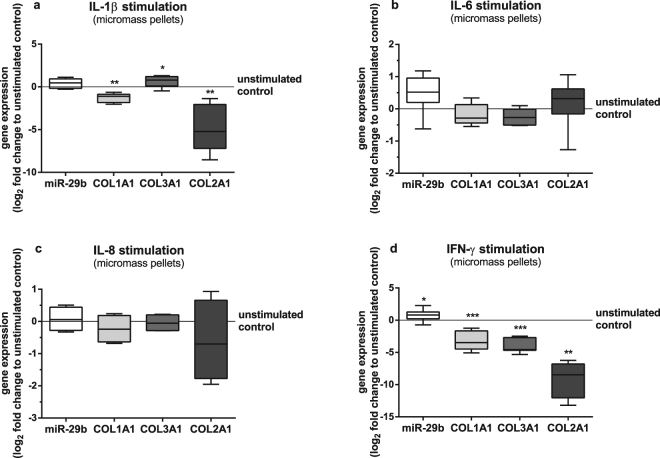
Gene expression of BMSC stimulated with different cytokines. miR-29b (white bars), *COL1A1* (light grey bars), *COL3A1* (dark grey bars) and *COL2A1* (black bars) gene expression of BMSC kept in micromass pellets and stimulated with (**a**) IL-1β (1 ng/ml; n = 8), (**b**) IL-6 (5 ng/ml; n = 6), (**c**) IL-8 (10 ng/ml; n = 4) and (**d**) IFN-γ (50 ng/ml; n = 11) for 7 days compared to unstimulated controls (zero line). *COL2A1* expression was only detected in 4 out of 11 cultures stimulated with 50 ng/ml IFN-γ. Results are expressed as box plots with median, the 25^th^ and 75^th^ percentiles and whiskers showing the largest and smallest values. * = p < 0.05; ** = p < 0.01; *** = p < 0.001; non-parametric Wilcoxon signed rank test for paired analysis.

Supernatants of BMSC in coculture with OA cartilage contain higher concentrations of pro-inflammatory cytokines like IL-1β, IL-6 and IL-8 than BMSC kept in monoculture^15^. These factors can have an inhibitory effect on collagen gene expression and synthesis in BMSC. As miR-29b expression is induced by soluble factors derived from OA cartilage, we investigated the potential of IL-1β, IL-6 and IL-8 to stimulate the expression of miR-29b. Stimulation of BMSC cultured as micromass pellets in chondrogenic medium with IL-1β caused no upregulation of miR-29b however, significantly reduced both *COL1A1* and *COL2A1* but significantly increased *COL3A1* gene expression compared to unstimulated BMSC (Fig. 7a). Stimulation with IL-6 or IL-8 did not regulate gene expression of miR-29b, *COL1A1*, *COL3A1* and *COL2A1* (Fig. 7b,c). Additionally, stimulation with LIF and TNF-α had also no effect on miR-29b expression (data not shown), but stimulation with IFN-γ led to significant upregulation of miR-29b and downregulation of *COL1A1* and *COL3A1* gene expression (Fig. 7d). IFN-γ downregulated *COL2A1*gene expression profoundly resulting in only 4 out of 11 samples with acceptable CT values in qPCR analysis. Titration with increasing concentrations of IFN-γ provoked increasing upregulation of miR-29b and increasing downregulation of *COL1A1*, *COL3A1* and *COL2A1* gene expression (Supplementary Figure S3).

Statistical data from all experiments (all figures including supplementary figures) are listed in Supplementary Table S5.

## Discussion

To develop MSC-based cartilage repair strategies for OA treatment, signals from the diseased microenvironment at the site of cell implantation need to be considered which affect transplanted cells’ ability to survive, to undergo chondrogenic differentiation and to maintain the stable phenotype of mature articular chondrocytes. We have previously demonstrated that OA cartilage influences chondrogenic differentiation of BMSC and composition of newly formed matrix *in vitro*, however without determining specific responsible factors^15^.

MiRs play a crucial role in RNA silencing and post-transcriptional regulation of gene expression in many different biological processes including differentiation in order to modulate and maintain normal physiological conditions. Different cell types and tissues express distinct sets of miRs which critically change during tissue differentiation or pathogenesis of diseases like OA. Several studies identified miRs which were differentially expressed between undifferentiated and chondrogenically differentiated MSC. Han *et al*. detected 4 miRs (miR-130b, -152, -26b, -28) which were upregulated in differentiated human BMSC compared to undifferentiated BMSC^31^. In human adipose-derived stem cells expression of miR-490-5p was gradually downregulated following induction of chondrogenic differentiation^36^. Complex regulatory miR networks are also involved in pathogenesis and progression of OA. MiR profiling of patient-derived osteoarthritic articular cartilage/chondrocytes in comparison to normal cartilage/chondrocytes revealed several differentially expressed miRs. For example, miR-576-5p was downregulated and miR-483-5p was upregulated in OA chondrocytes compared to normal chondrocytes^37^. Iliopoulos *et al*. identified 16 miRs differentially expressed in OA- compared to normal articular cartilage (upregulated miRs: miR-483, -22, -377, -103, -16, -223, -30b, -23b, -509; downregulated miRs: miR-29a, -140, -25, -337, -210, -26a, -373)^38^.

Our present study indicated for the first time a role for miR-29b in regulating collagen expression during chondrogenic differentiation of BMSC in the presence of OA cartilage. In BMSC cocultured with OA cartilage explants or cultured in medium conditioned by OA cartilage, expression of miR-29b-3p was significantly higher whereas *COL1A1* and *COL3A1* expression was significantly lower compared to BMSC kept in monoculture. Gain- and loss-of-function experiments with miR-29b mimic and inhibitor confirmed that higher miR-29b level caused lower *COL1A1* and *COL3A1* gene expression in BMSC. In BMSC transfected with a miR-29b inhibitor, we detected higher *COL1A1* and *COL3A1* expression level when cultivated in chondrogenic medium conditioned by OA cartilage compared to BMSC transfected with a non-targeting control miR. We concluded that soluble factors released by OA cartilage caused elevated miR-29b expression level leading to reduced collagen I and III levels without compromising collagen II.

Besides its inhibitory effect on collagen expression, miR-29b seems also play a role in cell survival and cell growth. MiR-29 family members affect proliferation and apoptosis of several different cell types. Of note, miR-29 promotes apoptosis by targeting the mRNA of anti-apoptotic proteins like Mcl-1 and Bcl-2^39,40^ and also inhibits proliferation^41,42^. Apoptosis is affected by regulating the expression of pro- or anti-apoptotic proteins of the Bcl-2 family. It was reported that miR-29b directly binds to the 3′-UTR of Mcl-1 and Bcl-2^39^. Both anti-apoptotic proteins are located in the outer mitochondria membrane which promotes cellular survival by inhibiting pore formation of pro-apoptotic proteins like Bax and Bak and thus preventing permeabilisation of the mitochondria membrane and cytochrome C release. Culturing BMSC in medium conditioned with OA cartilage led to higher miR-29b expression and reduced *BCL2* expression whereas *MCL1* gene expression level are equivalent to those of BMSC cultured in non-conditioned medium. However, overexpression of miR-29b by mimic transfection reduced both *BCL2* and *MCL1* expression significantly. Transfection of BMSC with a miR-29b inhibitor and subsequent culture in conditioned medium rescued *MCL1* gene expression. We thus conclude that higher miR-29b level induced by factors released from OA cartilage promotes apoptosis of BMSC indicated by higher caspase-3/7 activity in part by directly inhibiting the expression of regulators of apoptosis like the anti-apoptotic proteins Bcl-2 and Mcl-1. As Chen *et al*. reported, miR-29b can promote apoptosis of rat chondrocytes also by targeting progranulin leading to a changed ratio of Bcl2 to Bax and an increased protein level of cleaved caspase-3^43^. Contrary, miR-29b is not involved in regulation of proliferation of BMSC cultured with OA cartilage conditioned medium which was induced by soluble OA cartilage factors.

Next, we set out to identify factors which are responsible for the upregulation of miR-29b expression in BMSC during chondrogenic differentiation. Using medium conditioned by OA cartilage indicated that soluble factors released from OA cartilage explants are mainly responsible for the upregulation of miR-29b expression. Different mechanisms are known to regulate the expression of miRs which affect either their biosynthesis/maturation or their activity^44,45^. The biosynthesis of miR genes is regulated at multiple levels: pre-transcriptional by epigenetic control through DNA methylation^46^ or histone modifications^47^, at transcriptional level by transcription factors^48^, during processing of the primary miR transcript to the mature miR^44^ and post-transcriptional by RNA methylation, uridylation or adenylation^45^. Also, the activity of miRs is regulated by multiple factors affecting Argonaute loading or RISC formation with other proteins, and finally its turnover or degradation is controlled by nucleases^49^.

Not much is known about specific factors or mechanisms regulating miR-29b biosynthesis. In humans, two genes are described (MIR29B1 on chromosome 7 and MIR29B2 on chromosome 1) encoding two precursors (miR-29b-1 and miR-29b-2) which are spliced to mature miR-29bs of identical sequences^50^. The promoter regions of these genes contain different binding sites for several transcription factors like c-Myc or NF-κB which suppress expression of miR-29b^51^.

Le *et al*. observed that *SOX9* expression increased up to 7 days and decreased after 14 days. This pattern was inversely correlated with miR-29 expression in their MSC differentiation system to form cartilage discs meaning that overexpression of Sox9 in chondrosarcoma cells leads to a decrease in expression of the miR-29 family, whilst knockdown of Sox9 increased the expression^33^. As we did not detect changes in *SOX9* gene expression in BMSC in our culture system, we excluded Sox9 dependent regulation of miR-29b expression.

Factors related to inflammatory conditions are strong candidates for regulation of miR-29b synthesis as Le *et al*. reported that IL-1β increased the expression of miR-29b in chondrocytes^33^. In our previous study we observed that supernatants of BMSC cocultured with OA cartilage explants contained significantly higher IL-1β, IL-6 and IL-8 levels compared to monocultured BMSC^15^. However, stimulation of monocultured BMSC kept in micromass pellet with IL-1β, IL-6 and IL-8 did not induce upregulation of miR-29b. Also, stimulation with LIF, a member of the IL-6 family, which increased the expression of miR-29c via the JAK/STAT-3 pathway in rat kidney fibroblasts^52^ did not affect miR-29b expression in our culture regimen.

It is described that the miR-29a/b-1 promoter region contains several GAS-elements (IFN-γ activated sequences)^53,54^. IFN-γ activates the JAK/STAT pathway allowing activated STAT-1 dimers to bind to GAS-elements subsequently enhancing the expression of miR-29b in various melanoma cell lines^54^. Moreover, Tsuchida *et al*. detected IFN-γ in synovial fluid of OA patients but not in OA cartilage tissue extracts although OA chondrocytes produced IFN-γ^55^. In our experiments, IFN-γ stimulation significantly induced miR-29b expression and reduced *COL1A1* and *COL3A1* expression in a dose dependent manner compared to unstimulated BMSC indicating that IFN-γ is a critical regulator of miR-29b in BMSC during early chondrogenic differentiation.

In the present study, we characterized a critical role of miR-29b during chondrogenic differentiation of BMSC in an OA microenvironment. BMSC are able to undergo chondrogenic differentiation in our culture regimen indicated by increasing collagen II expression from day 7 to day 28 which seems to be not regulated via miR-29b. We suggest that miR-29b affects BMSC-based cartilage regeneration in an OA environment because the expression of collagen III, mainly expressed by undifferentiated MSC, and collagen I, a marker for dedifferentiated chondrocytes, are both inhibited by miR-29b which critically changes the composition of the newly formed ECM. In addition, higher miR-29b expression levels which have no influence on proliferation but promote apoptosis of BMSC might be considered as a control step preventing unfavourable excessive cell growth but might also have a negative effect if enhancing apoptosis of mature chondrocytes as described by Chen *et al*.^43^. A strong candidate for miR-29b regulation is IFN-γ, a factor produced by OA chondrocytes and detected in trauma cartilage extracts and synovial fluid of OA patients and healthy controls^55^. Without doubt, it is very likely that miR-29b expression *in vivo* is not induced by one single factor but by synergy of multiple factors. In that line, Roggli *et al*. demonstrated that stimulation with a mixture of different proinflammatory cytokines increased the expression of miR-29b in human pancreatic islet cells^56^.

We conclude that high miR-29b expression during chondrogenic differentiation of BMSC in an OA microenvironment has both supportive and possibly also unfavourable effects on BMSC-based OA cartilage regeneration. High miR-29b expression promotes apoptosis preventing excessive cell growth however also reducing the number of BMSC undergoing chondrogenesis. Furthermore, elevated miR-29b expression induced by OA cartilage inhibits the expression of collagen I and III without altering collagen II expression and thus alters the composition of newly formed ECM which might be critical to avoid formation of inferior fibrocartilage.

## Materials and Methods

### Ethical statement

Collection of human material was approved by the local ethics committee (Az: 14-101-0189; Ethikkommission, Universität Regensburg, email: ethikkommission@klinik.ukr.de) and with patients’ written informed consent. All experiments were performed in accordance with relevant guidelines and regulations. This manuscript does not contain information or images that could lead to identification of the tissue donors and for that I do not need any further personal consent.

### Isolation and culture of human BMSC, articular cartilage and subchondral bone explants

MSC were isolated from bone marrow which was flushed out of post-surgery discarded femoral heads of OA patients undergoing total hip replacement surgery and from pelvic bone material which accumulates when preparing the acetabulum for inserting the cup prosthesis. Bone marrow cells were separated by density gradient centrifugation according to established protocols^15,16^ and BMSC were expanded for three passages in StemMACS Expansion Medium (Miltenyi Biotec, Bergisch Gladbach, Germany). Due to low numbers of BMSC in the initial culture, cumulative population doubling level (CPDL) were counted after the P0 cell harvest. At each passage, BMSC were counted using a Cedex counter (Roche) and CPDL was determined by the following formula: CPDL = X + 3.322 (log N_H_ – log N_S_) where X is the PDL of the previous passage, N_H_ is the number of harvested cells and N_S_ is the number of seeded cells. The mean CPDL of BMSC after three passages was 5.66 ± 1.27 (n = 16) and the average time in expansion culture was 20.31 ± 2.18 days. This results in a mean proliferation rate of 0.28 ± 0.07 population doublings per day whereby the proliferation rate decreases from each passage to the next (Supplementary Figure S4). In total, BMSC from 39 different donors (mean age: 67.3 ± 9.1 years, range: 47–91 years, female: 68%) were used.

Human subchondral bone samples with articular cartilage were obtained from knee joints of OA patients undergoing total knee replacement surgery. Cartilage tissue was classified macroscopically as described previously^15,57^ and pieces of rather intact appearing cartilage regions were cut off from the subchondral bone. Round cartilage explants with a diameter of 8 mm were punched out with a biopsy punch (Stiefel, GlaxoSmithKline, Slough, UK). Any remaining cartilage was completely removed from subchondral bone which was cut into approximately 5 × 5 mm squares. Articular cartilage and subchondral bone explants were used for direct coculture experiments or for generation of conditioned medium. In total, cartilage from 40 different donors (mean age: 64.8 ± 8.0 years, range: 50–82 years, female: 55%) with similar OA grade and subchondral bone from 6 different donors (mean age: 61.8 ± 4.3 years, range: 57–68 years, female: 50%) were used.

### 3D coculture models: fibrin gels and micromass pellets

To investigate the influence of OA cartilage and bone on expression of collagen I, II, III and miR-29b in BMSC two different 3D coculture setups were used. For direct coculture, a previously described *in vitro* model was used^15,16^. Briefly, 1 × 10^6^ BMSC were resuspended in fibrinogen (100 mg/ml, Sigma-Aldrich, Steinheim, Germany), mixed with thrombin (5 U/ml, Baxter, Munich, Germany) and placed either on the surface of articular cartilage or subchondral bone explants (coculture) and as a droplet on the bottom of a 24-well plate (monoculture). After polymerization for 45 min at 37 °C, chondrogenic medium was added consisting of high glucose DMEM (Gibco, Thermo Fisher Scientific, Rochester, NY, USA) supplemented with 1% Antibiotic Antimycotic Solution, 0.1 µM dexamethasone, 40 µg/ml L-proline, 110 µg/ml sodium pyruvate, 50 µg/ml L-ascorbic acid 2-phosphate (all from Sigma-Aldrich), 1% ITS^+^-Premix (BD Biosciences, San Jose, CA, USA) and 10 ng/ml TGF-β3 (R&D Systems, Minneapolis, MN, USA). After 7 and 28 days, cells containing fibrin gels were scraped off the cartilage and bone explant surfaces (coculture) or off the well bottoms (monoculture) and used for RNA isolation. 6–8 independent BMSC and cartilage/bone donors were used (n = 6–8).

For indirect coculture, 2 × 10^5^ BMSC were centrifuged at 200 *g* for 5 min to a dense cell pellet in 96-well plates with conical bottoms (Nunc, Thermo Fisher Scientific). This micromass pellets were cultured in non-conditioned chondrogenic medium (NM) or in chondrogenic medium which was conditioned with one OA cartilage explant for 2 days (CM). After 7 days, 3 pellets were pooled for RNA isolation and pooled culture supernatants of 3 pellets were used for collagen isolation. Experiments with 7 different donors were analysed (n = 7).

### Overexpression and inhibition of miR-29b

6.5 × 10^4^ BMSC cultured in monolayer in 6-well plates were transiently transfected with 50 nM *mir*Vana miRNA mimic of hsa-miR-29b-3p (mimic), *mir*Vana miRNA Mimic Negative Control #1 (NT-miR), *mir*Vana miRNA inhibitor of hsa-miR-29b-3p (inhibitor) or *mir*Vana miRNA Inhibitor Negative Control #1 (NT-miR) (all from Ambion, Thermo Fisher Scientific) using Lipofectamine RNAiMAX Reagent (Invitrogen, Thermo Fisher Scientific) in Opti-MEM I Reduced Serum Medium (Gibco, Thermo Fisher Scientific).

Cells transfected with mimic/NT-miR were harvested after 48 h for RNA isolation and after 72 h for protein extraction. BMSC of 6 different donors were used (n = 6). BMSC transfected with inhibitor/NT-miR were trypsinised 24 h post-transfection, centrifuged to micromass pellets and cultured in CM for 7 days before RNA was isolated. This experiment was performed with BMSC from 8 different donors (n = 8).

### RNA isolation and gene expression analysis

Total RNA including the miRNA fraction was isolated with the MasterPure Complete RNA Purification Kit (Epicentre, Madison, WI, USA) according to the manufacturer’s protocol for tissue samples. RNA concentration was measured with the NanoDrop 2000 Spectrophotometer (Thermo Fisher Scientific) and RNA quality was analysed with the Agilent 2100 Bioanalyzer (Agilent Technologies, Santa Clara, CA, USA). For target gene expression analysis, 400 ng RNA was reversely transcribed into cDNA with the AffinityScript QPCR cDNA Synthesis Kit (Agilent Technologies). Quantitative real-time polymerase chain reaction (qPCR) was performed in duplicates using 20–50 ng cDNA, 200 nM self-designed primer according to Table 1 (Microsynth, Balgach, Switzerland) and Brilliant II SYBR Green QPCR Master Mix (Agilent Technologies) on Mx3005 P QPCR System (Agilent Technologies). For miR-29b expression analysis, 10 ng of total RNA was reversely transcribed with the TaqMan MicroRNA Reverse Transcription Kit using RT primers of TaqMan MicroRNA Assays for hsa-miR-29b-3p and U6 snRNA (Applied Biosystems, Foster City, CA, USA). qPCR was performed in duplicates using 1.33 µl of the appropriate cDNA, TM primers of TaqMan MicroRNA Assays and TaqMan Universal PCR Master Mix II, No AmpErase UNG (Applied Biosystems). Quantification of gene expression was performed according to the 2^−ΔΔCT^-method with normalization to GAPDH or U6. Expression levels are expressed as log_2_-fold changes relative to correspondent controls.

**Table 1:** Primer sequences for qPCR.

Gene	Primer sequence
*COL1A1*	fwd: 5′-ACGTCCTGGTGAAGTTGGTC-3′
	rev: 5′-ACCAGGGAAGCCTCTCTCTC-3′
*COL2A1*	fwd: 5′-TGCTGCCCAGATGGCTGGAGGA-3′
	rev: 5′-TGCCTTGAAATCCTTGAGGCCC-3′
*COL3A1*	fwd: 5′-CTTCTCTCCAGCCGAGCTTC-3′
	rev: 5′-TGTGTTTCGTGCAACCATCC-3′
*SOX9*	fwd: 5′-ACACACAGCTCACTCGACCTTG-3′
	rev: 5′-AGGGAATTCTGGTTGGTCCTCT-3′
*BCL2*	fwd: 5′-ATGTGTGTGGAGAGCGTCAA-3′
	rev: 5′-ACAGTTCCACAAAGGCATCC-3′
*MCL1*	fwd: 5′-AAGCCAATGGGCAGGTCT-3′
	rev: 5′-TGTCCAGTTTCCGAAGCAT-3′
*GAPDH*	fwd: 5′-CTGACTTCAACAGCGACACC-3′
	rev: 5′-CCCTGTTGCTGTAGCCAAAT-3′

### Protein extraction and Western Blotting

Transfected BMSC were lysed with Pierce RIPA buffer (Thermo Fisher Scientific) supplemented with Complete Mini Protease Inhibitor Cocktail (Roche Diagnostics, Mannheim, Germany) and sonicated three times (20 s, 5 cycles, 50% amplitude; SONOPULS Ultrasonic homogenizer, Bandelin, Berlin, Germany). Protein concentrations of these cell lysates and of cell culture supernatants obtained from BMSC kept as micromass pellets cultured in CM/NM were determined with Pierce BCA Protein Assay Kit (Thermo Fisher Scientific). Appropriate volumes of cell culture supernatants containing 1–5 mg total protein were digested with pepsin (5 mg/ml in 0.5 M acetic acid, 0.2 M NaCl; 100 µl/ml supernatant) for 48 h at 4 °C with rotation. Lysates were neutralized to pH 7.0 with 1 M Tris and pepsin-resistant fibrillar collagens were extracted by adding NaCl to a final concentration of 4.5 M and rotating overnight at 4 °C. Pelleted proteins were resuspended in precipitation buffer (0.1 M Tris, 0.4 M NaCl, pH 7.4) and collagens were precipitated with ethanol at −20 °C overnight, centrifuged and air-dried collagen containing pellets were resuspended in Laemmli buffer and denatured for 10 min at 95 °C. Denatured total protein from cell lysates (20 µg for collagen I detection, 40 µg for collagen III detection) and collagen lysates extracted from supernatants containing 1 mg total protein for collagen I and 5 mg for collagen III detection were separated in 8% polyacrylamide gels and transferred onto 0.2 µm PVDF membranes (Roche) at 90 mA for 4 h on ice. After blocking with milk, membranes were incubated with primary antibody at 4 °C overnight (anti-collagen I: Abcam #ab34710 (1:5000); anti-collagen III: Abcam #ab7778 (1:1000) or Santa Cruz #sc-514601(1:100)) and with secondary HRP-conjugated antibody (anti-rabbit: Jackson Immuno Research #711-036-152 (1:10000) or anti-mouse: Thermo Fisher Scientific #62-6820 (1:2000)) for 1 h at RT. Protein bands were detected with SuperSignal West Femto Maximum Sensitivity Substrate (Thermo Fisher Scientific) in the chemiluminescence imager Chemi-Smart 5000 (Peqlab Biotechnologie, Erlangen, Germany) with the software Chemi-Capt 5000 version 12.8 (Vilber Lourmat, Eberhardzell, Germany). Membranes with total protein lysates were stripped with ReBlot Plus Mild Antibody Stripping Solution (Merck Millipore, Darmstadt, Germany), subsequently incubated with anti-beta actin antibody (1:5000) overnight (Abcam #ab8227) and detected as described above. Quantification of band densities was performed with ImageJ 1.48 v (NIH, USA). Experiments with 6 different donors were analysed (n = 6).

### Caspase 3/7 activity assay (Apoptosis assay)

Caspase 3/7 activity was analysed with the Apo-ONE Homogenous Caspase 3/7 Assay (Promega, Madison, WI, USA). 4 × 10^3^ BMSC seeded in black 96-well plates with clear bottom (Falcon, Corning, NY, USA) were transfected with mimic/inhibitor/NT-miR or cultured in CM/NM. 96 h after mimic transfection or culture in CM and 72 h after inhibitor transfection and culture in CM, Apo-ONE Caspase-3/7 Reagent was added and fluorescence was measured 3–8 h later in a plate reader (Tecan GENios with Magellan 6; Tecan Group Ltd., Männedorf, Switzerland). Experiments were performed in triplicates. CM and BMSC of 8 different donors were used for each of the three experimental setups (n = 8).

### BrdU incorporation assay (Proliferation assay)

Cell proliferation was quantified with the colorimetric BrdU Cell Proliferation ELISA (Roche). 4 × 10^3^ BMSC/96-well were transfected with mimic/NT-miR or cultured in CM/NM. 72 h later, BrdU labelling solution was added and cells were incubated for 24 h at 37 °C and 5% CO_2_. Following the manufacturer’s instructions, after adding stop solution absorbance at 450 nm was measured. Experiments were performed in triplicates. CM and BMSC of 6–8 different donors were used for each of the two experimental setups (n = 6–8).

### Inhibition and Stimulation of miR-29b expression

BMSC micromass pellets were cultured in NM or CM supplemented with Complete Mini Protease Inhibitor Cocktail with and without EDTA (Roche) for 7 days to analyse if the upregulation of miR-29b could be prevented when proteases like matrix metalloproteases (MMP) are inhibited. BMSC of 6 different donors were used (n = 6).

To investigate the influence of different cytokines on miR-29b expression, BMSC micromass pellets were stimulated for 7 days with the following cytokines (all from Peprotech, Hamburg, Germany) in chondrogenic medium: IL-1β (1 ng/ml), IL-6 (5 ng/ml), IL-8 (10 ng/ml) and IFN-γ (1 ng/ml, 10 ng/ml and 50 ng/ml). BMSC of 4–11 different donors were used (n = 4–11).

### Statistical analysis

Statistical analyses and graphing were performed with GraphPad Prism version 6 (GraphPad Software, San Diego, CA, USA). Results are expressed as box plots with median, the 25^th^ and 75^th^ percentiles and whiskers showing the largest and smallest values. Non-parametric Wilcoxon signed rank test for paired analysis was used to test for significant differences compared relative to control values of 0 or 100%. p values < 0.05 were considered as statistically significant and are listed in Supplementary Table S5. Significant outliers (α = 0.05) were determined with the Grubbs test.

### Data availability statement

The datasets generated and analyzed during the current study are available from the corresponding author on reasonable request.

## Electronic supplementary material

Supplementary Figures S1-S4 and tables S5
