# Supplementary material for: miR-29b regulates expression of collagens I and III in chondrogenically differentiating BMSC in an osteoarthritic environment
Source: Sci Rep. 2017 Oct 16;7:13297. doi: 10.1038/s41598-017-13567-x (PMC5643533; doi:10.1038/s41598-017-13567-x)
Supplement: Supplementary file 1 — Supplementary Figures S1-S4 and tables S5 [file 41598_2017_13567_MOESM1_ESM.doc]

**miR-29b regulates expression of collagens I and III in chondrogenically differentiating BMSC in an osteoarthritic environment**

Ute Mayer^1,2^, Achim Benditz^2^, *Susanne Grässel^1,2^

^1^Dept. Orthopaedic Surgery, Exp. Orthopaedics, ZMB/Biopark 1, University of Regensburg, Germany

^2^Dept. Orthopaedic Surgery, Asklepiosklinikum, Bad Abbach, Germany

*Correspondence to Susanne.Graessel@klinik.uni-regensburg.de


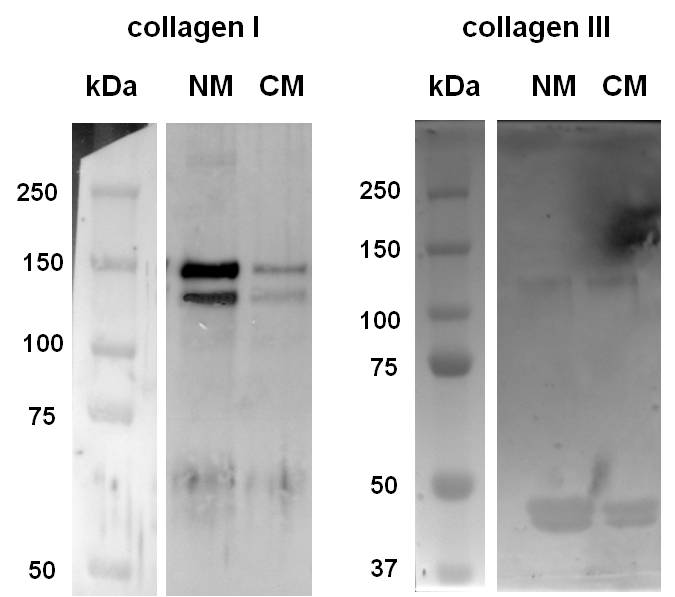


**Supplementary Figure S1: Full-length western blot images**

Representative collagen I and III full-length western blot images from collagen extracts of cell culture supernatants of BMSC kept in micromass pellets for 7 days in NM and CM.

CM: with OA cartilage conditioned medium; NM: non-conditioned medium.

**Supplementary Figure S2: Quantification of BrdU incorporation during DNA synthesis**

(**a**) Proliferation of BMSC in monolayer cultured in chondrogenic medium conditioned with OA cartilage (CM) relative to BMSC cultured in non-conditioned medium (NM, line at 100 %, n=6). (**b**) Proliferation of BMSC in monolayer transfected with miR-29b mimic relative to BMSC transfected with a non-targeting control miR (NT-miR, line at 100 %, n=8). Results are expressed as box plots with median, the 25^th^ and 75^th^ percentiles and whiskers showing the largest and smallest values. * = p<0.05; non-parametric Wilcoxon signed rank test for paired analysis. CM: with OA cartilage conditioned medium; NM: non-conditioned medium; mimic: miR-29b mimic; NT-miR: non-targeting control miR.


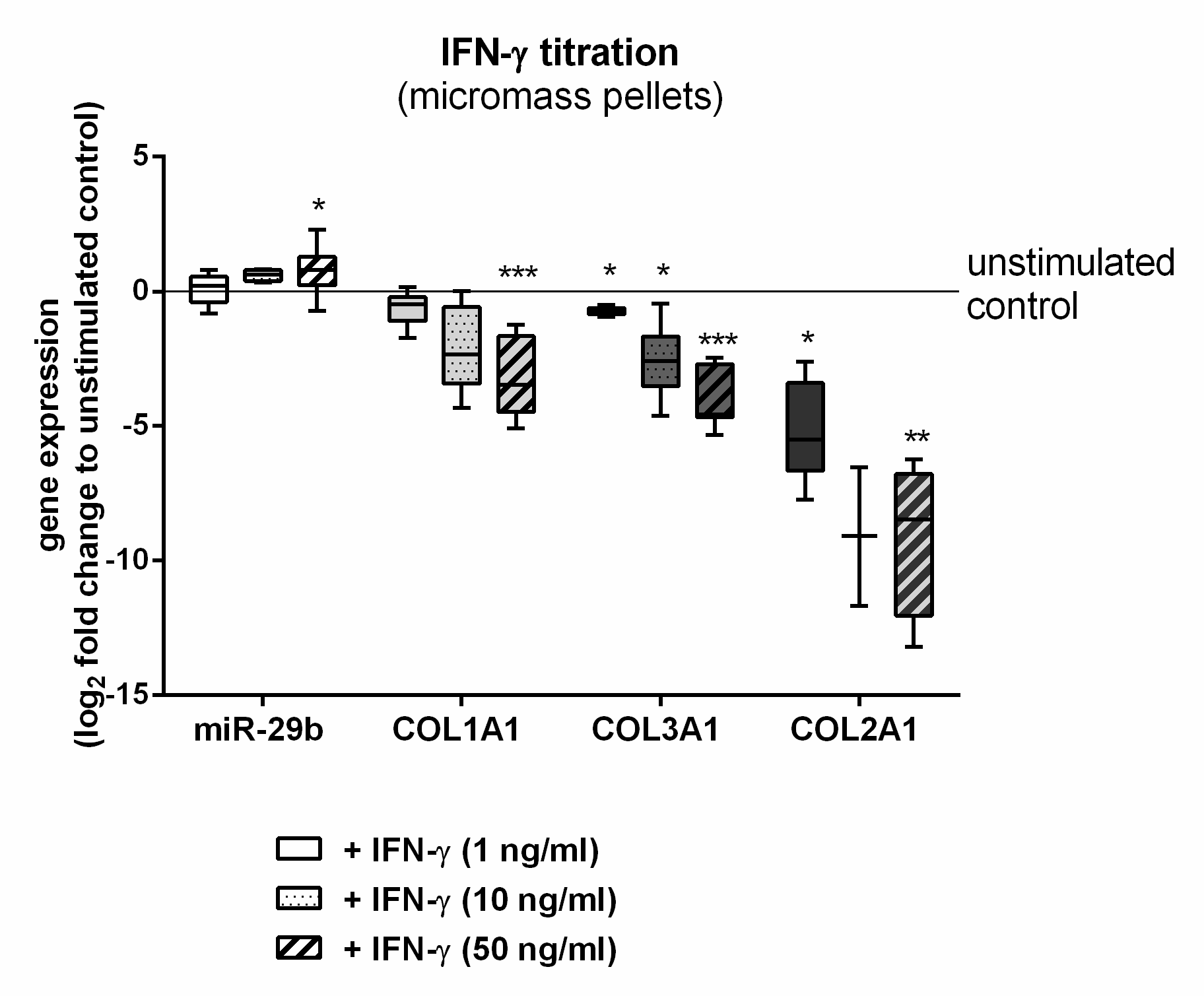


**Supplementary Figure S3: Gene expression of BMSC titrated with increasing concentrations of IFN-γ**

miR-29b (white bars), COL1A1 (light grey bars), COL3A1 (dark grey bars) and COL2A1 (black bars) gene expression of BMSC kept in micromass pellets titrated with increasing concentrations of IFN-γ (1 ng/ml, bars without pattern, n=6; 10 ng/ml, dotted bars, n=6; 50 ng/ml, hatched bars, n=11) for 7 days compared to unstimulated controls (zero line).

COL2A1 was detected in 5 out of 6 stimulations with 1 ng/ml, in 3 out of 6 with 10 ng/ml and 4 out of 11 experiments using 50 ng/ml IFN-γ.

Results are expressed as box plots with median, the 25^th^ and 75^th^ percentiles and whiskers showing the largest and smallest values.* = p<0.05, *** = p<0.001; non-parametric Wilcoxon signed rank test for paired analysis.

**Supplementary Figure S4: Cumulative population doubling level and proliferation rate of BMSC in expansion culture**

Due to low numbers of BMSC in the initial culture, cumulative population doubling levels (CPDL) were counted after the P0 cell harvest. Increasing CPDL was detected from P1 to P3 (**a**) and the mean CPDL of BMSC after three passages was 5.66 ± 1.27 and the average total time in expansion culture was 20.31 ± 2.18 days. This results in a mean proliferation rate of 0.28 ± 0.07 population doublings per day (**b**) whereby the proliferation rate decreases from each passage to the next.

Results are expressed as mean with SD (n=16). ** = p<0.01, *** = p<0.001; non-parametric Mann Whitney test for unpaired analysis.

**Supplementary Table S5: Statistical data (p-values)**

| **p-values of Fig. 1a** | miR-29b | COL1A1 | COL3A1 | COL2A1 |
| --- | --- | --- | --- | --- |
| coculture with OA cartilage day 7 vs. monoculture day 7 (n=8) | **0.0234** | **0.0078** | **0.0078** | **0.0078** |
| coculture with OA cartilage day 28 vs. monoculture day 28 (n=8) | **0.3828** | **0.0234** | **0.0547** | **0.7422** |
| **p-values of Fig. 1b** | miR-29b | COL1A1 | COL3A1 | COL2A1 |
| coculture with OA bone day 7 vs. monoculture day 7 (n=6) | **0.4375** | **0.1563** | **0.0938** | **0.2500** |
| coculture with OA bone day 28 vs. monoculture day 28 (n=6) | **0.6875** | **0.0625** | **0.0938** | **0.0938** |
| **p-values of Fig. 1c** | miR-29b | COL1A1 | COL3A1 | COL2A1 |
| monoculture day 28 vs.  monoculture day 7 (n=8) | **0.7422** | **0.25** | **0.4609** | **0.0078** |
| coculture with OA cartilage day 28 vs. coculture with OA cartilage day 7 (n=8) | **0.2188** | **0.1484** | **0.9453** | **0.0078** |
| coculture with OA bone day 28 vs. coculture with OA bone day 7 (n=6) | **0.6875** | **0.8438** | **0.8438** | **0.0625** |
| **p-values of Fig. 1d** | SOX9 |  |  |  |
| coculture with OA cartilage day 7 vs. monoculture day 7 (n=6) | **0.4375** |  |  |  |
| coculture with OA cartilage day 28 vs. monoculture day 28 (n=6) | **1.0000** |  |  |  |
| coculture with OA bone day 7 vs. monoculture day 7 (n=6) | **0.8438** |  |  |  |
| coculture with OA bone day 28 vs. monoculture day 28 (n=6) | **0.6875** |  |  |  |
| **p-values of Fig. 1e** | SOX9 |  |  |  |
| monoculture day 28 vs.  monoculture day 7 (n=6) | **0.2188** |  |  |  |
| coculture with OA cartilage day 28 vs. coculture with OA cartilage day 7 (n=6) | **0.0625** |  |  |  |
| coculture with OA bone day 28 vs. coculture with OA bone day 7 (n=6) | **1.0000** |  |  |  |

| **p-values of Fig. 2a** | miR-29b | COL1A1 | COL3A1 | COL2A1 |
| --- | --- | --- | --- | --- |
| CM vs. NM (n=7) | **0.0156** | **0.0156** | **0.0156** | **0.4375** |
| **p-values of Fig. 2b** | Collagen I | Collagen III |  |  |
| CM vs. NM (n=4-6) | **0.0313** | **0.2500** |  |  |

| **p-values of Fig. 3** | miR-29b | COL1A1 | COL3A1 | COL2A1 |
| --- | --- | --- | --- | --- |
| (**a**) Mimic vs. NT-miR (n=6) | **0.0313** | **0.0313** | **0.0313** |  |
| (**c**) Inhibitor+CM vs. NT-miR+CM (n=8) | **0.0078** | **0.0391** | **0.0156** | **0.2969** |

| **p-values of Fig. 4** | Caspase 3/7 activity |
| --- | --- |
| (**a**) CM vs. NM (n=8) | **0.0313** |
| (**b**) Mimic vs. NT-miR (n=8) | **0.0156** |
| (**c**) Inhibitor+CM vs. NT-miR+CM (n=10) | **0.0098** |

| **p-values of Fig. 5** | miR-29b | BCL2 | MCL1 |
| --- | --- | --- | --- |
| (**a**) CM vs. NM (n=7) | **0.0156** | **0.0156** | **0.375** |
| (**b**) Mimic vs. NT-miR (n=6) | **0.0313** | **0.0313** | **0.0313** |
| (**c**) Inhibitor+CM vs. NT-miR+CM (n=8) | **0.0078** | **0.0156** | **0.9453** |

| **p-values of Fig. 6** | miR-29b | COL1A1 | COL3A1 |
| --- | --- | --- | --- |
| (**a**) CM+protease inhibitor with EDTA  vs. NM (n=6) | **0.0313** | **0.0313** | **0.0313** |
| (**b**) CM+protease inhibitor without EDTA  vs. NM (n=6) | **0.0625** | **0.0313** | **0.0313** |

| **p-values of Fig. 7** | miR-29b | COL1A1 | COL3A1 | COL2A1 |
| --- | --- | --- | --- | --- |
| (**a**) IL-1β stimulation  vs. unstimulated control (n=8) | **0.1953** | **0.0078** | **0.0391** | **0.0078** |
| (**b**) IL-6 stimulation  vs. unstimulated control (n=6) | **0.2188** | **0.3125** | **0.0938** | **0.4375** |
| (**c**) IL-8 stimulation  vs. unstimulated control (n=4) | **0.8750** | **0.6250** | **0.6250** | **0.3750** |
| (**d**) IFN-γ stimulation  vs. unstimulated control (n=11) | **0.0137** | **0.001** | **0.001** | **0.01** (n=4) |

| **p-values of Supplementary Fig. S2** | BrdU incorporation |
| --- | --- |
| (**a**) CM vs. NM (n=6) | **0.0313** |
| (**b**) Mimic vs. NT-miR (n=8) | **0.5469** |

| **p-values of Supplementary Fig. S3** | miR-29b | COL1A1 | COL3A1 | COL2A1 |
| --- | --- | --- | --- | --- |
| IFN-γ (1 ng/ml) vs.  unstimulated control (n=6) | **0.6875** | **0.0625** | **0.0313** | **0.05**  (n=5) |
| IFN-γ (10 ng/ml) vs.  unstimulated control (n=6) | **0.4375** | **0.0625** | **0.0313** | (n=3) |
| IFN-γ (50 ng/ml) vs.  unstimulated control (n=11) | **0.0137** | **0.001** | **0.001** | **0.01**  (n=4) |

| **p-values of Supplementary Fig. S4b** | proliferation rate |
| --- | --- |
| P1 vs. P2 (n=16) | **0.0680** |
| P1 vs. P3 (n=16) | **0.001** |
| P2 vs. P3 (n=16) | **0.0019** |
